# A mixed-methods study of health worker migration from Jamaica

**DOI:** 10.1186/s12960-016-0125-8

**Published:** 2016-06-30

**Authors:** Gail Tomblin Murphy, Adrian MacKenzie, Benjamin Waysome, Joan Guy-Walker, Rowena Palmer, Annette Elliott Rose, Janet Rigby, Ronald Labonté, Ivy Lynn Bourgeault

**Affiliations:** WHO/PAHO Collaborating Centre on Health Workforce Planning and Research, Faculty of Health Professions, Dalhousie University, 5869 University Avenue, Halifax, Nova Scotia B3H 4R2 Canada; Ministry of Health, Kingston, Jamaica; Faculty of Medicine, University of Ottawa, Ottawa, Ontario Canada; Telfer School of Management, University of Ottawa, Ottawa, Ontario Canada

**Keywords:** Dental auxiliaries, Doctors, Health workers, Human resources for health, Jamaica, Midwives, Migration, Nurses, Policy

## Abstract

**Background:**

This study sought to better understand the drivers of migration, its consequences, and the various strategies countries have employed to mitigate its negative impacts. The study was conducted in four countries-Jamaica, India, the Philippines, and South Africa-that have historically been ‘sources’ of health workers migrating to other countries. The aim of this paper is to present the findings from the Jamaica portion of the study.

**Methods:**

Data were collected using surveys of Jamaica’s generalist and specialist physicians, nurses, midwives, and dental auxiliaries, as well as structured interviews with key informants representing government ministries, professional associations, regional health authorities, healthcare facilities, and educational institutions. Quantitative data were analyzed using descriptive statistics and regression models. Qualitative data were analyzed thematically. Multiple stakeholder engagement workshops were held across Jamaica to share and validate the study findings and discuss implications for the country.

**Results:**

Migration of health workers from Jamaica continues to be prevalent. Its causes are numerous, long-standing, and systemic, and are largely based around differences in living and working conditions between Jamaica and ‘destination’ countries. There is minimal formal tracking of health worker migration from Jamaica, making scientific analysis of its consequences difficult. Although there is evidence of numerous national and international efforts to manage and mitigate the negative impacts of migration, there is little evidence of the implementation or effectiveness of such efforts. Potential additional strategies for better managing the migration of Jamaica’s health workers include the use of information systems to formally monitor migration, updating the national cadre system for employment of health personnel, ensuring existing personnel management policies, such as bonding, are both clearly understood and equitably enforced, and providing greater formal and informal recognition of health personnel.

**Conclusion:**

Although historically common, migration of Jamaica’s health workers is poorly monitored and understood. Improved management of the migration of Jamaica’s health workers requires collaboration from stakeholders across multiple sectors. Indeed, participating stakeholders identified a wide range of potential strategies to better manage migration of Jamaica’s health workers, the implementation and testing of which will have potential benefits to Jamaica as well as other ‘source’ countries.

## Background

Jamaica is one of the largest countries in the Caribbean, with an estimated population of 2.7 million [1]. Life expectancy is estimated at 72.7 years [2], with infant and under-five mortality rates of 16 and 18 per 1,000, respectively [3]. Jamaica’s GDP is roughly US$ 14 billion, of which public expenditure on healthcare is approximately 5 % [4]. According to WHO, in 2003 (the most recent year for which data were available), Jamaica’s physician density per 1,000 population was 0.85, while its nursing/midwifery and its dentist density were 1.67 and 0.08, respectively [3]. Jamaica has two medical schools offering a 4-year medical program and eight nursing schools offering training at the certificate, diploma, and baccalaureate levels [5, 6]. Jamaica’s healthcare system is funded from public and private sources in roughly equal measure [4]. The public healthcare system is administered by the Ministry of Health (MoH) and four regional health authorities (RHAs).

Jamaica has one of the highest emigration rates in the world [7]. Approximately 750,000 Jamaicans emigrated between 1970 and 2003 [8]-roughly 22,000 people per year. The top destination for Jamaicans has historically been the United States, with more Jamaicans migrating there than to all other countries combined [8]. In this context, the migration of highly trained health professionals, such as physicians and nurses, is an ongoing issue facing Jamaica as well as other Caribbean countries [2, 9]. Research conducted by the Caribbean Community and Common Market (CARICOM) in 2006 estimated that the Caribbean had lost in excess of 50,000 nurses over the preceding 10 years, equivalent to a monetary loss of training expenses of US$ 2.2 million [10]. In 2009, the World Bank estimated that the number of CARICOM-trained nurses practicing abroad was roughly three-fold to those practicing in the CARICOM [10].

Although a considerable amount of research has been performed on the migration of health professionals to or from individual countries, the factors that influence migration have not been compared across countries. In addition, existing research on the migration of health professionals has focused almost exclusively on physicians and nurses, with little consideration of the many other types of healthcare professions. There has also been little study of the various policies and programs implemented in attempts to reduce migration from source countries [11].

The work described herein forms part of a study that sought to inform ‘source’ and ‘destination’ country policies pertaining to health worker migration by addressing the knowledge gaps described above. This was performed by exploring the consequences of the migration of highly skilled health workers from participating ‘source’ countries, specifically India, Jamaica, the Philippines, and South Africa, to Canada and other destination countries including the United States, the United Kingdom, and Australia. The study focused on issues related to the migration of physicians and nurses specific to each country, as well as for two other professions of particular interest to each country. In the case of Jamaica, the country of interest for the present study, the migration trends of physicians, nurses, midwives, and dental nurses/auxiliaries are assessed. The overall study addresses (1) the present picture of, and recent historic trends in, the migration of highly skilled health personnel in the Philippines, India, South Africa, and Jamaica; (2) the most critical consequences, according to those ‘on the ground’, of the emigration of highly skilled health workers and the evaluation of these consequences to optimize the potential for comparative policy analyses; and (3) the range of program and policy responses that have been considered, proposed, and implemented to address these critical causes and consequences as well as the outcomes of these responses.

The aim of the present article is to describe the methods and findings of the Jamaica component of the study.

## Methods

The study used a mixed-methods, comparative approach, drawing on information from scoping reviews of published literature, surveys of health workers, and key informant interviews in each country.

### Study coordination

The project activities in Jamaica were coordinated through the Jamaica MoH, where a joint country research team (‘the team’), made up of representatives from the Ministry’s Policy and Planning and Strategic Human Resource Management departments as well as the Canadian researchers at Dalhousie University and the University of Ottawa, was established. A Project Coordinator was also contracted in Jamaica to organize and execute local research activities on behalf of the team.

### Scoping review

A scoping review of published literature on health professional migration from Jamaica was conducted using a process consistent with the approach described by Arksey and O’Malley [12]. The search strategy targeted three key concepts: migration, health professionals, and Jamaica. A search of peer-reviewed literature was performed on May 9, 2012, using Ovid MEDLINE® from 1946 to that date. The searches employed MeSH terms for the concepts of ‘migration’ and ‘health professionals’ as well as the term ‘Jamaica’. Non-peer-reviewed literature, including publicly available reports, publications, proceedings of meetings, memos, and correspondence between various stakeholder groups involved in the migration of health professionals, was also searched. This search began by targeting key organizational websites such as the Global Health Workforce Alliance, the Organisation for Economic Co-operation and Development (OECD) documents on migration of health workers, the World Bank, and WHO. The websites of governmental and intergovernmental organizations in the Caribbean, such as the Pan American Health Organization (PAHO), MoH Jamaica, and CARICOM, were also searched for relevant policy documents addressing each of the research questions.

Inclusion and exclusion criteria were developed to eliminate studies not relevant to the research questions. The majority of the collected articles were published after 2000; hence, analysis was restricted to these more recent documents. Articles not related to either Jamaica specifically or the Caribbean in general were excluded, as were those that related to Jamaica but did not focus on the migration of health professionals. A standardized literature extraction tool was developed, piloted and refined through the review process to ensure a consistent approach to the documentary analysis and synthesis. Subsequent to this extraction, the Global Health Observatory and World Bank databases were accessed again to update the population and health statistics for Jamaica.

### Instrument development

Two instruments were developed by the research team to collect data from key stakeholders in each country. These were an anonymous survey for members of each of the four professions being studied (physicians, nurses, midwives, and dental auxiliaries in the case of Jamaica), and a guide for interviews with key informants such as policymakers and representatives of professional associations (see Sampling and Recruitment below). Several survey questions were adapted from those used by Connell [13] and Anarfi et al. [14] for earlier studies of health worker migration. Key questions addressed various aspects of respondents’ views on migration, the factors they identify as encouraging them to migrate, as well as specific steps they may have taken toward migrating. The main key informant interview questions were designed to seek participants’ perspectives on the causes, consequences and policy responses to health worker migration; some interview questions were adapted from those used by the Global Health Workforce Alliance [15].

### Research ethics

Approval to conduct the study using the developed instruments was obtained from the research ethics boards at the Jamaica MoH, the University of Ottawa, and Dalhousie University prior to data collection. Informed consent was obtained from all interviewees and survey respondents prior to their participation.

### Sampling and recruitment

#### Key informant interviews

To complement the ‘front line’ perspectives of practicing health workers, the more system-oriented perspectives of several key informants were obtained through a set of interviews. These key informants were representatives of several public and private sector organizations in Jamaica, including government ministries, health professional regulatory bodies, health professional associations, national development agencies, private sector healthcare facilities, public sector healthcare facilities, recruiters, and academic institutions.

Three distinct criteria guided the selection of key informants: (1) their organization’s role in migration-related issues, (2) their position in the organization, and (3) their experiences relating to the migration of highly trained health personnel. A list of potential key informants was agreed upon by the team based on these criteria. Formal invitations were sent via email to 25 individuals. Each email included an official study invitation, study consent form, contact details for project focal points in both countries, and the list of interview questions. Different questions were prepared for each type of organization listed above. All those invited to participate in the interviews agreed to do so; the interviews were conducted by the Project Coordinator.

#### Survey of health workers

In Jamaica, the survey targeted four professional groups-physicians, nurses, midwives, and dental auxiliaries. The survey was made available in two formats-a paper version and an electronic, online version administered through Dalhousie University’s Opinio web platform. Accurate listings of licensed members of these professions with contact information were not available for sampling; therefore, members of the four professions being studied were invited to participate in the online survey through several methods-promotional flyers and bookmarkers were provided to professional associations and councils for distribution to their membership, printed copies of these items were sent to major public and private hospitals in Kingston for wider distribution and posting on notice boards, and packages of printed invitations were also sent to professional conferences and meetings for distribution. Each invitation included the URL for the survey, the survey deadline, and the Project Coordinator’s contact information. Planned visits were made to hospitals in Kingston to encourage participation and a survey corner was set up at the country’s two largest hospitals-Kingston Public Hospital (KPH) and the Victoria Jubilee Hospital (VJH)-with a computer to prompt potential respondents to complete surveys while on a break.

To complement the online survey process, packages of printed questionnaires, along with envelopes in which to enclose completed copies, were distributed to public hospitals and parish health departments for distribution within their facilities and/or health districts. The major hospitals in St. Catherine, Kingston and St. Andrew parishes (KPH, VJH, University Hospital of the West Indies, and Spanish Town Hospital) were again visited during the questionnaire distribution phase to encourage participation. Distribution stations were set up at strategic points within these facilities to ensure the engagement of health workers entering and leaving the facilities as well as those on duty. Printed sheets containing details about drop-off were issued with each survey package. Large drop-off boxes were also posted at these facilities and collection arrangements were made with select staff members at each facility and department. All completed questionnaires were checked by the Project Coordinator and temporarily stored in a secured area in the Ministry of Health prior to being packaged and sent via registered mail to Dalhousie University for data entry and analysis.

### Data collection and analysis

A total of 27 key informant interviews were conducted between April and July 2013; 25 were conducted in person and two via telephone. In all instances, participants opted to be interviewed in the privacy of their offices or in a private meeting room at their organization. Each organization was represented by one or two persons and all interviews were recorded for accuracy using a digital voice recorder. Interview transcripts were subsequently prepared and distributed to the interviewees for their review and validation prior to being sent to Dalhousie for coding and analysis.

All recorded and transcribed interviews were coded and thematically analyzed using NVivo 10 software and coding categories determined by the research team. The purpose of this analysis was to identify common themes across the responses given by individual participants and to address the first and second research questions related to the consequences of health worker migration as well as the current and possible future policy and program responses.

Over the study period, 361 completed surveys were obtained from representatives of the four health professions (47 % nurses, 19 % physicians, 12 % dental auxiliaries, 11 % midwives, and 11 % nurse-midwives) being studied. Of these, the majority (*n* = 322) were completed using the paper version of the survey and the remainder (*n* = 39) were completed using the online platform. Data from the paper surveys were entered into the same electronic data file housing the online survey data for descriptive and regression analyses using SAS 9 software. The purpose of these analyses was mainly to address the first of the study’s research questions by investigating the degree to which respondents are considering migrating and the steps they may have taken toward doing so, and to estimate the degree to which these views and actions are explained by respondent traits such as age, sex or profession.

### Validation of findings

Once preliminary findings were obtained, several activities were undertaken to ensure their validity within Jamaica and across the other countries being studied. The first of these was a stakeholder engagement forum held at the University of the West Indies, Mona Campus, in September 2013. The purpose of the forum was to share the preliminary findings with key Jamaican stakeholders and discuss the degree to which they were consistent with these stakeholders’ experiences. Participants in the forum represented a wide range of Jamaican organizations involved in healthcare education, planning and service delivery across the country, including six government ministries and agencies, the four RHAs, the two largest private healthcare facilities in the country, members of each of the four included professions, and the country’s largest health professional education institution. The forum used profession-specific group sessions and interactive presentations to stimulate dialogue. The participants in the forum confirmed that the study’s preliminary findings were consistent with their experience within the Jamaican context.

The next validation activity was a 2-day workshop held in Ottawa in September 2013 at which representatives from each of the participating countries shared and discussed the challenges and successes encountered in implementing the study in their respective countries; it was noted by multiple participants at this workshop how similar many of the challenges experienced were across each country.

The final validation activity was a pair of broader engagement workshops held with representatives of Jamaica’s four RHAs in January 2014; the first of these was held in Kingston for representatives of the Southern and South East RHAs and the second was held in Ocho Rios for representatives of the Western and North East RHAs. The main purpose of these workshops was to discuss the implications of the study findings for health workforce planning and policymaking in Jamaica. These implications are described in the next section.

### Limitations

The study was limited by several factors. Chief among these was the inadequacy of Jamaica’s existing information systems to keeping accurate counts of the health workers currently in Jamaica and systematically tracking health worker migration. Without such information, it is not possible to fully assess the scope of Jamaican health worker migration or to rigorously evaluate its determinants. These limitations also made it impossible to systematically reach out to Jamaican health workers who have already migrated so as to include their perspectives in the study. Another limitation is the study’s cross-sectional nature, which precludes the possibility of longitudinal analysis. In addition, the lack of accurate data on the number of health workers currently living in Jamaica means the response rate to the health workers’ survey cannot be accurately estimated; responses from a larger number of health workers would have strengthened the study.

## Results and Discussion

The results of the scoping review and analyses of survey and key informant interview data are presented below according to the study’s research questions.

### Who is migrating?

Neither Jamaica’s government nor its health professional associations or regulatory bodies formally track the migration of health workers from the country, making a comprehensive analysis of this phenomenon problematic. Knowledge of the state of health worker migration is therefore limited to sporadic estimates from cross-sectional studies. The most recent estimates found during the scoping review suggested that 50 % of all physicians trained in Jamaica since 1991 have emigrated [16], while two thirds of nurses ever trained in Jamaica have emigrated [17, 18]. In 2009, the World Bank also estimated that the number of CARICOM-trained nurses practising abroad was three-fold that practicing within the CARICOM region [9]. The same report identified Canada, the United States, and the United Kingdom as popular destinations, with more than 1,800 Caribbean nurses migrating to these countries between 2002 and 2006.

**Fig. 1:**
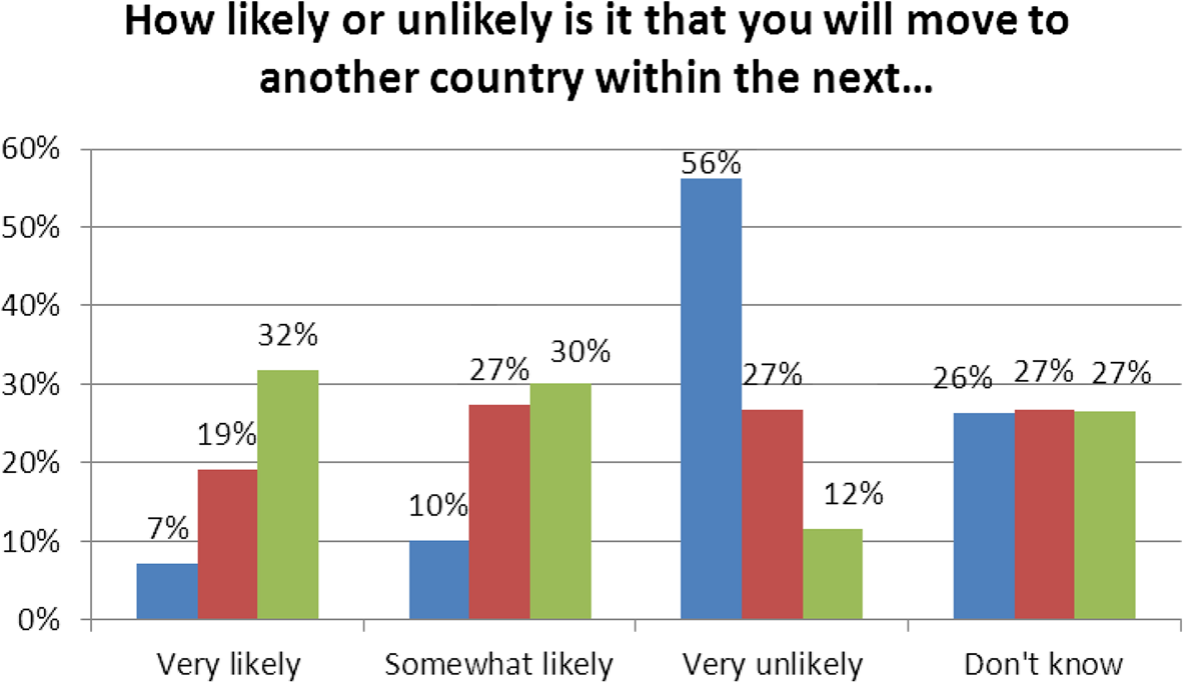
Self-reported likelihood of migrating within different time intervals. Blue, 6 months; Red, 2 years; Green, 5 years

Survey respondents were asked how much consideration they had given to migration. Most (79 %) reported that they had given it some or a great deal of thought. Respondents were also asked whether their level of interest in migrating had changed in the past 5 years; 35 % reported that their interest in migrating had increased, whilst 50 % reported that it had stayed the same and 15 % that their interest had decreased. Respondents were also asked how likely it was that they would migrate within the next 6 months, 2 years, or 5 years. Although most respondents indicated that it was very unlikely that they would leave within the next 6 months, most also reported it was very likely or somewhat likely that they would do so within the next 5 years (Fig. 1).

**Fig. 2:**
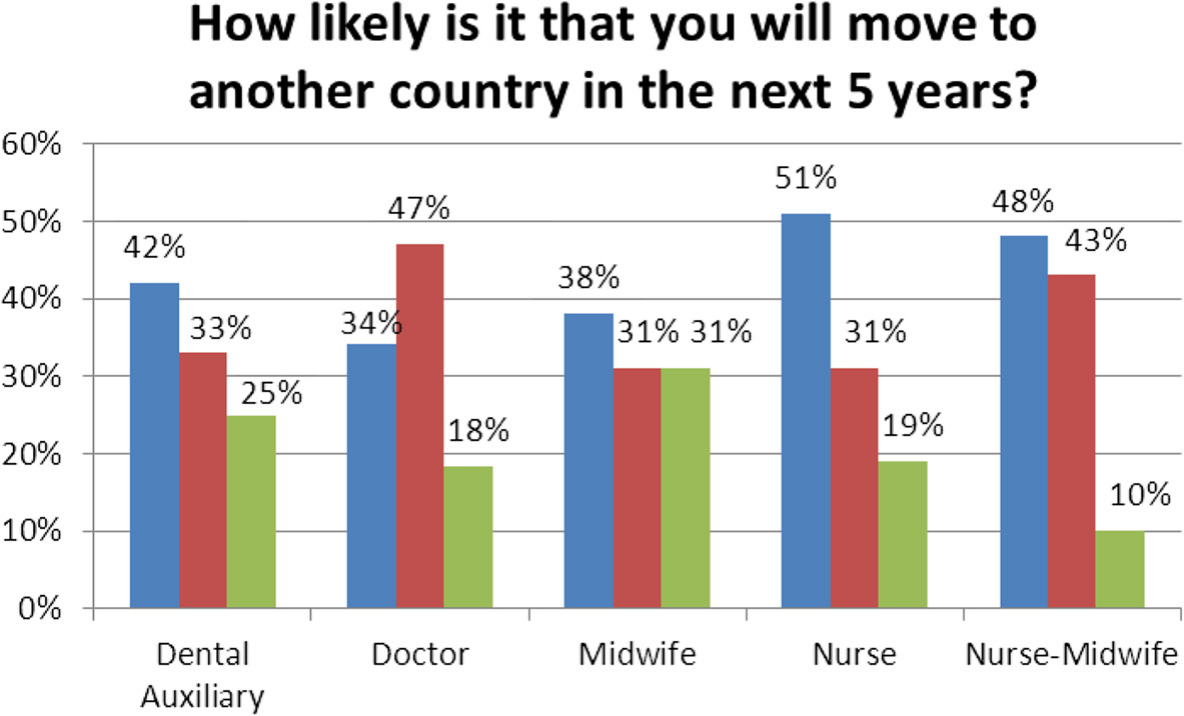
Self-reported likelihood of migration within 5 years by profession. Blue, very likely; Red, somewhat likely; Green, unlikely

Direct entry midwives reported the least likelihood of migration within the next 5 years, while registered nurses and dual-trained midwives reported the highest (Fig. 2). This was consistent with information obtained through key informant interviews. A higher proportion of female respondents (31 %) compared to males (23 %) reported that they were very likely to migrate within the next 5 years, and a higher proportion of respondents aged 25–34 (48 %) reported that they were very likely to migrate within the next 5 years compared to other age groups. However, none of these differences-by profession, age group or sex-was statistically significant.

A logistic regression model was developed to assess the degree to which respondents’ self-reported likelihood of migrating within the next 5 years (‘very likely’ vs. ‘somewhat likely’ or ‘not very likely’) was explained by any of several factors including their age, sex, profession, years in practice, the type of training institution they attended (publicly vs. privately funded), the main funding source for their training (personal or family funds, bank loan, scholarship/bursary, NGO), and main sector of work (public vs. private; Table 1). When each of these factors was controlled for, the only significant factor in predicting respondents’ intention to migrate was age, with older respondents reporting a lower likelihood of migrating. Regression models of respondents’ intent to migrate within 6 months and 2 years, and of the frequency with which respondents reported considering migration, yielded nearly identical results.

**Table 1:** Logistic regression of self-reported likelihood of migration within 5 years

Coefficient	Odds ratio	Confidence interval	*P* value
Profession-Doctor	0.77	0.27–2.21	0.626
Profession-Dental nurse/auxiliary	1.33	0.35–5.05	0.674
Profession-Midwife	2.21	0.65–7.52	0.205
Profession-Registered nurse/midwife	0.36	0.09–1.35	0.129
Years in profession	1.05	0.98–1.13	0.167
Private/public training institution	1.05	0.55–1.99	0.889
Some training costs paid through local scholarship	1.00	0.99–1.01	0.552
Some training costs paid through NGO	0.99	0.96–1.03	0.602
Some training costs paid through bank loan	1.00	0.98–1.01	0.720
Work mainly in public sector	0.59	0.09–3.93	0.589
Sex	0.66	0.20–2.17	0.498
Age	1.13	1.06–1.22	0.001

### Why are they migrating?

The scoping review found few Jamaica-specific studies of how and why health workers migrate. Nevertheless, there was considerable evidence from the Caribbean as a whole, and the factors identified as driving migration of health workers from the Caribbean include better working conditions in destination countries [9, 19]; better quality of life in general [19, 20]; burnout [19, 21, 22]; better wages [9, 20, 23]; better pension schemes, benefits, and opportunities for professional development and career advancement [9, 19, 24]; more effective healthcare systems [9, 19, 24]; reunification of families [20, 25]; favorable immigration policies [20]; and crime and violence [26, 27]. Although most of these studies did not compare the relative importance of these factors, analysis by the World Bank [9] identified better wages as the most important driver of migration of Caribbean nurses.

Within the present study, Jamaican survey respondents were asked to identify the three most important reasons for wanting to migrate within two categories-living conditions and working conditions. The three highest-ranking reasons within each category are shown in Table 2. The highest-ranking reason for a desire to migrate was related to respondents’ income (chosen by 64 % of respondents) relative to costs of living (chosen by 57 % of respondents), consistent with Caribbean-wide studies [9, 20, 23]. Related to this point, respondents were also asked to describe their current economic situation, with possible responses being Excellent, Good, Fair, or Poor; only 15 % of respondents described their current economic situation as ‘Good’ or better (fewer than five respondents described it as ‘Excellent’), whereas 62 % described it as fair and 23 % as poor. The second highest-ranking reasons for a desire to migrate were related to the quality of infrastructure within their workplaces and within the country as a whole. As one interviewee noted:

**Table 2:** Top-rated reasons for wanting to migrate

Category	Reason
Working conditions	1. Your income compared to what you would like to earn (64 %)
	2. The state of infrastructure where you work (11 %)
	3. Lack of opportunity for further education/advancement (6 %)
Living conditions	1. High cost of living (57 %)
	2. Quality/upkeep of public infrastructure (8 %)
	3. The ability to obtain good quality consumer goods (5 %)

“*When you look at the situation a lot of people think it is about the salary. It is not just about the salary; the working conditions must be borne in mind.*”

Another added:

“*What affects migration are the conditions at work. Money is a big thing, but your setting is also important - so the conditions at work, in your environment and so on. This is a big thing that is driving migration… you will find that this affects rural to urban …*[migration]*, and, in terms of external, from developing to developed countries, because the conditions are such that you can get what you want to use and you don’t have to be fighting to compromise.*”

The prominence of lacking opportunities for further education or advancement as a driver of migration among respondents was consistent with Caribbean-wide findings from previous studies [9, 19, 24]. Participants in stakeholder engagement forums noted, however, that education opportunities are distinct from advancement opportunities, and that the latter are those more lacking in Jamaica. Related to this point, several interviewees identified Jamaica’s current cadre system for staffing as a driver of migration. The cadre is the set of established posts for all health workers in the public sector, identifying the number and type of health workers that may be employed in each government-run healthcare facility and healthcare role. The current cadre has not been updated or amended in over 40 years to account for the changes in population demographics, population health needs, practice patterns, service models, or any other changes that have occurred during that time. Therefore, the cadre was repeatedly described as being outdated and inadequate. Specifically, the current cadre is perceived as having an insufficient number of permanent posts for all types of health workers required to cover the healthcare needs of the Jamaican population. As one interviewee opined:

“*The cadre of 1974 is obsolete. Our population has grown 30–40 % more and we are still using that existing cadre so we have never budgeted adequately for health care, not to mention for nurses.*”

Another wondered:

“*How can we worry about migration when we are not providing jobs for the persons trained? It makes no sense. There is a tremendous need in the public sector for more persons to work but there is not a commensurate availability of posts in the hospital system, thus leaving persons with two choices-to either go to the private sector or migrate to the Caribbean, Europe or North America.*”

In an attempt to meet these needs, the RHAs are limited to employing large numbers of personnel on a temporary basis, which provides little job security for either the employee or the employer, and does not allow for career advancement. Moreover, there is extensive reliance on services provided on a ‘sessional’ or overtime basis, which come at an increased cost to the system. Unfortunately, Jamaica’s health information systems do not allow for the systematic tracking of the use of these employment arrangements. As one interviewee noted:

“*We have quite a few staff who are employed temporarily and they are temporary over a lengthy period. So that does create a little bit of dissatisfaction amongst the staff because they cannot be appointed-there are no posts to put them in and that is one of the challenges that we face.*”

Survey respondents were also asked whether they had experienced any periods of unemployment over the previous 5 years; 22 % of respondents reported that they had, although no statistically significant differences were found in this value between professions. This is consistent with anecdotal reports of health worker unemployment from key informants. One interviewee, in addressing the issue of nurses’ unemployment, reported:

“*Well, initially there was not a problem with employment but now there is. I have been getting many calls. Persons who graduated* [8 months ago] *are still not employed. Just before you came one person called and…. it was the same thing that she was saying to me. Twenty-five of them that graduated from a university and they were told that after the budget presentation and when they went back they were told they were not hiring but only replacing people who have retired and so they are still waiting to be employed. So we still have nurses sitting at home despite the shortages at hospitals.*”

Violence and crime have also emerged as one of the factors influencing migration of skilled health professionals. One interviewee advised, “*The security is another push factor because nobody is immune.*” Another recounted, “*I remember when they killed the man at* [redacted] *hospital; I was sitting on my ward the day when the gunshots were blazing across the place when they went on… to kill the patient.*” Psychological and physical violence toward health workers are widespread in Jamaica [26]; for example, most physicians and nurses at KPH reported experiencing threats of violence from patients or family members [27]. Concerns about violence and crime have been identified drivers of migration beyond the health sector [28] and Jamaica’s homicide rate is among the highest in the world [29].

### How are they migrating?

**Fig. 3:**
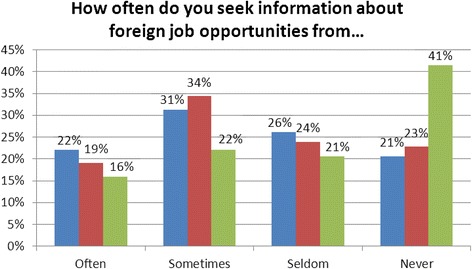
Sources of information on foreign job opportunities. Blue, newspapers or professional journals; Red, personal contacts abroad; Green, recruitment websites

The survey included several questions regarding the various mechanisms that may facilitate the migration process. Respondents indicated that they received inquiries about working abroad more often from colleagues in other countries than from recruitment agencies. In addition to being contacted, most respondents reported that they have also sought information themselves with regards to migration from a variety of sources, including newspapers, professional journals, recruitment websites, and personal contacts (Fig. 3). Both of these results are consistent with findings by the World Bank indicating that migration patterns in general are partly facilitated by social networks of expatriates in the destination country [9]. The same study also noted that most Jamaican nurses surveyed cited the inability to access information on migration as the sole reason they have not yet done so. One interviewee stated that:

“*You can’t just get up and migrate like that because if you don’t have papers filed by qualified family, if you don’t have papers to work overseas, you can’t just go. You have to be recruited; it is a long process and it is not easy unless you are recruited. So that is probably the main reason why we don’t see more because I am sure if tomorrow a recruiter came here from Florida or New York, and say they wanted to interview people who want to migrate, I am sure there would be some nurses and I am sure the meeting room would be full.*”

Respondents were also asked to state, given that they intended to migrate, their preferred destination country. The most frequently cited destinations (some respondents identified more than one country) across all professions were Canada (cited by 64 % of respondents), the United States (45 %), another Caribbean country (8 %), and the United Kingdom (6 %). These responses are similar to findings from a recent OECD study, where the most popular destinations for Jamaican physicians were identified as the United States, the United Kingdom, and Canada [30]. This is also consistent with the interview data. One interviewee disclosed:

“*Most of the doctors that come to us are transient, OK? They have finished their studies at UWI* [University of the West Indies]*, gone out and done their internship and they decide to specialize. They do not plan to specialize at UWI-they are going to the US or Canada. And they look around and say ‘Where can I get a half-way decent salary that I can support my family, not have to do a lot of extra sessions because I want to study for the overseas exams?*”

The survey also included questions about concrete steps respondents may have taken toward migration; 10 % of respondents reported that they had applied for a work permit in a foreign country, 12 % for resident status, and 33 % had applied to write the licensing exam for their profession in another country. Despite a lack of knowledge on whether any of these various applications were successful, the results suggest that a substantial portion of the highly-trained health workers who participated in the survey were very serious with regards to leaving Jamaica to practice in another country.

### Return migration

The survey also included questions about return migration for respondents who may have spent time practicing in another country but have since returned to Jamaica. Forty respondents (11 % of the sample) reported having worked as a member of their health profession in another country, most commonly in another Caribbean country. Among return migrants, 58 % reported that they had worked in another country for 3 years or less, and 77 % said they had the same professional status while working abroad as they did in Jamaica. Overall, 33 % of respondents said they had experienced problems upon their return, such as adjusting to a lower salary relative to the cost of living, poorer infrastructure, equipment, and supplies, and being treated with hostility or disrespect as a result of having migrated. Perhaps related to this point, 53 % of return migrants reported that they were not sure whether their return was permanent or not. Several interviewees had comments on the subject:

“*Some do* [return] *but it is the minority. Once people go abroad and they spend any time abroad it is much less likely that they will come back. Sometimes they intend to come back but if their children start to go to school or anything like that then…* [they stay].”

“*I don’t know of many who actually come back. Occasionally so, few and far between, you may meet professionals who, and we call them returners when they come back to the system… It becomes an issue where procedurally things are done a little different here from how they would do it there. Those in England seem to be sticklers for the formality of everything and that seems to go against the brain of the local persons.*”

“*Usually they come back with an accent. They come back with an accent so that alone alienates them from everybody else which is so unfortunate. You will hear them say ‘the English Nurse’, but she is Jamaican.*”

### What are the impacts of health worker migration?

While it is difficult to measure the impacts of such migration due to the lack of formal tracking or monitoring systems, the scoping review identified some evidence on three distinct levels of impact: system, provider and patient/population levels. The system level encompasses health professional education, infrastructure, and population health initiatives. The provider level, on the other hand, includes recruitment and retention, while the patient/population level speaks directly to the quality of care and remittances received.

#### System level

The literature suggests that investment in nursing education in the Caribbean has not yielded the expected returns due to migration. According to a United Nations Secretariat report, the migration of nurses’ resulted in a loss of US$ 15–20 million invested by Caribbean countries in training them [21]. Yan [22] and Jones et al. [25] reported similar findings, estimating that in 2000 and 2003, the Caribbean region experienced a loss of US$16.7 million and US$ 13.5 million, respectively, in the public investment of training nurses.

The shortage of nursing tutors in the English-speaking CARICOM region is also one of the impacts of migration highlighted by the World Bank [9]. The report notes that the high student-to-nurse tutor ratio is an impediment to the development of local nursing programs and that the shortage of nursing tutors is leading to a situation where tutors are being ushered into teaching before attaining the relevant qualifications.

One interviewee in Jamaica noted:

“*We have lost a whole generation of nurses. Our records show that every health facility lose their qualified staff on a continuous basis, so at this time there are a few seniors at the top and the bottom is filled with juniors. We have lost the middle. So we are not able in most places to realize our succession plan. This is a critical time for the profession of nursing if the gap between academia and service is to be closed. Tutors are short. So are the nurse managers as those who coach and mentor the nurses*.”

Another interviewee stated:

“*In terms of… those who leave as mid-career, late-career nurses, this limits the pool of educators you can recruit from. That certainly does impact in terms of the quality of teachers that you have, their level of experience and expertise, and the quality of education and training that you can offer to the undergraduate and graduate students particularly in the clinical areas.*”

The reduced supply of nurses in the health sector also affects the implementation of care and prevention strategies for a large portion of the population affected by the HIV/AIDS virus [17, 18]. A shortage in nursing staff is often linked to the fall in immunization coverage experienced in Jamaica in the late 1990s and early 2000s; the proportion of the population immunized against poliomyelitis decreased from 90 % in 1997 to 81 % in 2003, whereas the immunization rates for measles and diphtheria, pertussis, and tetanus dropped from 88 % and 90 % in 1997 to 79 % and 81 % in 2003, respectively [31]. In this vein, a joint PAHO-CARICOM report highlighted the need to strengthen the public health infrastructure, improve public health training, and invest in public health research towards more evidence-based decision making in the Caribbean [32].

#### Provider level

Migration has also increased the challenges associated with the recruitment and retention of health professionals. A 2001 PAHO analysis (cited by Jones et al. [25]), shows that between 1998 and 2000, the migration of Caribbean nurses resulted in increased recruitment and retention costs to Caribbean countries. In Jamaica, the government has attempted to address the shortage of health professionals by recruiting from Cuba and Nigeria on a temporary basis [33]. As one interviewee stated, “*The population is growing and we are expanding our health facilities and we are expanding the areas of specialization, yet we are not retaining our highly skilled staff*.”

Several key informants described challenges associated with recruiting and retaining health professionals in the rural areas of the country, particularly physicians. This has led to the contracting of a large number of Indian and Burmese physicians to work in the rural parts of the country. One interviewee noted, “*Doctors in the urban areas are hesitant in coming to the rural areas. They will work in the urban areas like Kingston, St. Andrew readily rather than come out.*” There are also challenges with providers having dual practice in both the public and private systems in Jamaica to supplement their public sector incomes or moving entirely to the private sector. One interviewee said:

“*Because we do lose people-and we are mindful of the fact that the government salary scale has not been competitive-and so whilst we have people, we have trained some, we have given some scholarships to be trained, but our retention is not at the level we would want it to be because people sometimes move to the private sector to earn more money, and they also move out of the country for the same reason.*”

#### Patient/population level

The shortage of health professionals in Jamaica [34, 35] is made worse by migration. Such shortages have been found to increase workload and therefore burnout among those health workers who remain in the country. One study estimated the costs associated with burnout-related sick days among Jamaican nurses at US$ 2.6 million per year [22]. This problem is worsened because health workers who migrate tend to be those with experience, reducing the quality of care available to patients and the sources of leadership and mentoring available to professionals who remain [9].

**Fig. 4:**
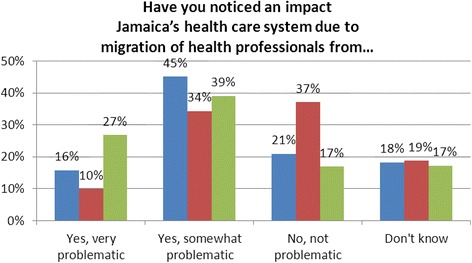
Perceived impacts of different types of migration. Blue, rural to urban areas; Red, public to private sector; Green, Jamaica to other countries

Survey respondents were asked whether they had noticed any problems with Jamaica’s healthcare system due to any of three types of migration-from rural to urban areas, from the public to the private sector, and from Jamaica to other countries (Fig. 4). Respondents viewed both rural to urban and international migration of health workers to be problematic for Jamaica.

As one interviewee reported, “*As people move from country to town, it means that you have less human resources in the rural areas to man your clinics and to man your health centres and all of that.*”

Another major impact of migration for Jamaica is the remittances sent back by Jamaicans working abroad. Remittances from all Jamaicans living abroad (i.e. not only health workers) are valued at an estimatd US$ 2 billion per year, which amounts to one seventh of Jamaica’s GDP [4]. Related to this point, the vast majority (80 %) of survey respondents reported that they would send money home in the event that they left Jamaica to work elsewhere. Several interviewees reported that Jamaica has been benefiting from various gifts donated by Jamaican health workers who have migrated. While the total value of these contributions is difficult to quantify due to the lack of a formal gift registry, it has been noted that they have been helping to address various needs and resource gaps:

“*So let us say, Nurse X and Doctor X have migrated… what they do is not only give supplies and pharmaceuticals and send them back to the country but they do volunteer their time and their skills. So they come and set up medical missions especially in far reaching rural areas where our system itself can’t sometimes manage.*”

### What strategies have been implemented or considered to mitigate these impacts?

The scoping review identified several domestic and multinational strategies described in published documents as having been developed to better manage the migration of health workers and/or to mitigate any of its negative impacts (Table 3).

**Table 3:** Past and current strategies to mitigate health worker migration consequences

Scope	Strategy
Global	• Health Worker Migration Initiative/WHO Global Code of Practice on International Recruitment of Health Workers
Caribbean-wide	• Managed Migration Program o Nurses Homecoming Program o Health and tourism model o Caribbean-Canadian program • Return of Qualified Nationals program
Jamaica-destination countries	• Circular migration program (Jamaica-United States of America)
National	• Bonding • Increased public sector wages • Increased enrolment, updated curricula, increased professional development for nurses • Other improved incentives

Two major initiatives at the global and regional levels have been developed, one on international recruitment codes and one to manage migration at the global level. The Health Worker Migration Initiative [36] is a task force implemented in 2007 to provide recommendations to WHO for the development of the WHO Global Code of Practice on the International Recruitment of Health Workers, adopted at the 63rd World Health Assembly in 2010 [37, 38].

At the Caribbean level, the Managed Migration Program[17, 18] emerged as a response to the large loss of Caribbean health professionals to destination countries, aiming to manage migration through several initiatives formed as unilateral/bilateral or multilateral policies [9]. In the Caribbean, there were several country-specific programs in the region. Under the Homecoming Program, in 2003, Caribbean nurses who practiced abroad were invited back to their home countries to volunteer and share their skills with local nurses [17, 18, 22, 39]. The health and tourism model attempted to recruit nurses from destination countries, such as the United States, Canada and the United Kingdom, to practice in the Caribbean for up to 6 months within the year, emphasizing the potential appeal of a healthier work-life balance from working in the Caribbean. Under this program, recruited nurses were to receive the same rate of compensation as Caribbean nationals for their employment in the Caribbean. An advertisement for the program was placed in one of the British nursing journals and 30 nurses responded to the advertisement in one day; however, no data exists on whether these respondents were actually employed in the Caribbean [17, 18, 22]. The Caribbean-Canadian program was designed as a 12-month, multi-stakeholder initiative to curb the negative effects of nurse migration from the Caribbean by offering post-graduate placements in Canada for specialized training; students would then return to Jamaica to practice [40]. The Return of Qualified Nationals Programme implemented by the International Organization for Migration was designed to facilitate the return migration of health professionals and other professions from Canada, the United States and the United Kingdom to Jamaica, with the stipulation that participants have an offer of employment from their home country and would thus contribute to its development [24]. The Jamaican Nursing Council has implemented an initiative through which Jamaican nurses have the ability to practice alternately in the United States and Jamaica. It was believed that the close proximity of the United States would permit nurses to practice there for 2 weeks per month while working in Jamaica the rest of the month, thus promoting circular migration. Airfare and accommodation costs were to be the responsibility of the nurses. The goal of this program was to provide the opportunity for nurses to acquire additional skills in the United States and earn more income while reducing outright migration of nurses from Jamaica [17, 18, 22].

Jamaica’s Ministry of Health has implemented several policies designed to achieve, among other things, greater retention of highly-trained health workers, not only within Jamaica, but within the public sector. These include a bonding or return-of-service program in which health workers whose training is financed by the government of Jamaica agree to provide a term of public service after completing their training; increased wages for workers in the public sector; increased enrolment and redesigned curricula for nurses’ pre-licensure training; and increased post-licensure professional development opportunities for nurses.

What each of these initiatives has in common is that the scoping review found no evidence of any analysis of their impacts, if any, on health worker migration or its consequences. Further, with the exception of the Jamaican policies, there is little or no documentation available to show the degree to which they have even been implemented. Recent comments from Jamaica’s Minister of Health, however, suggest that the country remains concerned about the degree to which ‘destination’ countries are complying with the WHO Global Code of Practice [41].

A number of potential strategies for the better monitoring and management of the migration of Jamaica’s health workers were suggested by study participants. For example, many expressed the view that increased remuneration for health workers in Jamaica would help reduce migration to other countries or to the private sector. Others suggested that non-financial incentives, such as recreation facilities, housing, and child care programs for staff, would help with retention. However, participants from within the MoH noted that Jamaica’s limited public funds have necessitated cuts to government spending across multiple sectors, and that nearly 90 % of the health sector budget is already devoted toward remuneration, therefore leaving little potential to obtain funds for such incentives by drawing either from other sectors or other areas of the health portfolio such as supplies or infrastructure.

Several potentially inexpensive strategies to better manage health worker migration from Jamaica were suggested by study participants. The most immediate of these was to share the findings of the study with Jamaica’s Ministries of Health and Finance as a means of informing dialogue between these partners regarding national health priorities. Among others were potential changes to the way health personnel are managed, including a long-awaited update to the cadre system, which was described by numerous stakeholders as a barrier to effective management of health workers, specifically, and health system performance more broadly, because it is not sufficiently flexible to allow for either the permanent employment of needed personnel or career progression of personnel. A revised cadre that provides the number and type of permanent posts for the number and type of health workers actually required to meet the changing health needs of Jamaica as well as the professional needs of its workers therefore has great potential to improve the satisfaction and morale of the health workforce, not to mention the overall performance of the healthcare system in terms of meeting the population’s healthcare needs.

Participants also noted that the dedication and commitment of many of Jamaica’s health workers not only to providing quality healthcare but also to contributing to an improved healthcare system are tremendously valuable national resources that must be protected and cultivated. As one interviewee reported:

“*There are still those who find fulfilment in service. They feel a sense of commitment to the nation-that this is where they are from, this is where they have benefitted from training and they feel that the government has provided them with opportunity for employment and they want to give back… I want to console myself in thinking that the majority of persons who are here are people who feel a sense of commitment.*”

Related to this was the suggested provision of greater formal and informal recognition for hard work and dedicated service by members of the health workforce. Regarding formal recognition, it was noted by several participants that even small tokens, such as certificates of appreciation, can go a long way toward making people feel valued. In terms of informal recognition, it was suggested that a display of greater ‘emotional intelligence’-that is, making greater efforts to understand and address health workers’ motivations, needs, and concerns-by health workforce managers and other personnel would help health workers to feel more valued and thus less likely to migrate.

It was also reported that many Jamaican health workers perceive some existing human resource management policies as being implemented in an inconsistent and/or non-transparent manner. These include polices pertaining to selection of personnel for advancement, promotion, or education leave, bonding of new graduates, the hiring of foreign-trained vs. Jamaican-trained personnel, and the allocation of other resources such as housing. Efforts to ensure that these policies are more clearly understood by Jamaica’s health workers and implemented consistently and effectively therefore have the potential to improve the effectiveness of the healthcare system while also improving the satisfaction of its personnel. Further, several participants suggested that the option for persons who have received education funding from the MoH in return for a ‘bonded’ term of service to pay off these bonds early be removed.

Related to the issue of the clarity and transparency of policies, several participants noted that communication between the MoH and health workers is not perceived as being optimal, and that this should be kept in mind when attempting to plan and implement any kind of change. It was also noted, however, that while there may be room for the MoH to improve its policy mechanisms and/or their content, it is incumbent on Jamaica’s health workers themselves to ensure that they devote adequate effort to informing themselves on Ministry policies and programs.

Regardless of the strategies implemented to better manage the migration of Jamaica’s health workers, there is a need for greater monitoring capacity on an ongoing basis. Establishing such a capacity will require the MoH to collaborate with several partners, such as the various Professional Councils and Associations governing individual health professions as well as the country’s Passport, Immigration and Citizenship Agency, in order to obtain accurate counts of the number of licensed personnel living in Jamaica as well as the number of those migrating and those returning and to facilitate ongoing monitoring. This will allow for the evaluation of strategies implemented to better manage the migration of Jamaica’s health workers, whilst facilitating Jamaica’s ongoing compliance with the principles of the WHO Global Code on the International Recruitment of Health Personnel pertaining to domestic human resources for health management.

While there would be planning and change management costs associated with developing and implementing any of these suggested strategies, these are likely to be minimal in comparison to those required for other suggestions such as increasing salaries or providing additional housing. Further, there is also the potential for the strategies described above to reduce some personnel costs. For example, an updated and more responsive cadre system could allow more personnel to be hired on a permanent basis, reducing the need for reliance on ‘sessional’ workers who are paid at a premium rate. One interviewee described an example:

“*The sessionals are at a premium compared to what a full time nurse’s salary will be, so the less that you have to use sessionals the better for your bottom line at the hospital. So you would want to be able to keep and retain qualified nursing staff to avoid using sessionals too often throughout the year.*”

More broadly, participants in the validation workshop reported that improved staffing and management structures would contribute to reduced turnover among Jamaica’s health workers and, by extension, a reduction in the direct (e.g. advertising for replacement personnel) and indirect (e.g. lost productivity while replacement personnel get ‘up to speed’) costs associated with those losses. There would also be benefits in the retention of a greater portion of human capital-the experienced personnel who form the backbone of Jamaica’s healthcare system not only by providing quality patient care but by serving as leaders and mentors for other personnel so as to further strengthen the system throughout their careers.

The data collected also point to the fact that professionals are leaving because they can. While the factors influencing their decision are many, there is apparently no real restriction to their efforts to leave the public service, or the country. Hence, many experienced professionals have migrated, leaving the health sector financially worse off and more human resource deficient. The findings of this study suggest the number of Jamaican health workers who migrate will only continue to grow, further exacerbating this problem. Additionally, those leaving include workers who signed bonding agreements but have left without completing their agreed-upon terms of work in Jamaica. One suggested means of addressing the issue of unfulfilled bonds was greater collaboration between Jamaica’s Ministry of Finance, the Passport, Immigration and Citizenship Agency, and the MoH to ensure travel restrictions on those health workers who have signed bonding agreements with the government of Jamaica. Restrictions suggested by participants included the holding of professional certificates/degrees until bonds have been paid up and increasing the cost of migration-related paperwork. Results from the survey, however, suggest that the latter restriction may not be as effective as participants might hope. Respondents were asked whether an increase in the fees charged by the government for emigration documents would make them more or less likely to emigrate, or would have no impact on their decision to migrate; 64 % reported that such a fee increase would have no impact on their decision to migrate, 9 % reported that it would make them more likely to do so, 13 % reported it would make them less likely to migrate, and 14 % reported being unsure.

## Conclusion

Evidence emerging from this study has confirmed that nurses, midwives, physicians and dental auxiliaries are taking concrete steps toward leaving Jamaica for other countries, albeit in different numbers and frequency. The study also identified that this outward movement is strongly influenced by many long-standing and systemic issues highlighted in other publications/studies such as income relative to costs of living, lack of education and career opportunities, and undesirable working conditions and infrastructure. Related to these issues, the prospects of better income, increased opportunities, and a better quality of life in destination countries were identified as factors influencing the decision to migrate-these factors are common in other countries. The issue of the MoH’s outdated personnel cadre for the health sector, the associated lack of permanent employment or opportunities for advancement, and the perceived effect of encouraging health worker migration is perhaps more unique to Jamaica. Because the migration of Jamaica’s health workers is not systematically tracked, it is not possible to precisely measure its scope or impacts. That said, data gathered as part of this study identified a mix of positive (e.g. the contribution of remittances from abroad) and negative (e.g. reducing the number of experienced health workers available in Jamaica) impacts. Investment in administrative databases that allow for the systematic measurement of health worker migration from Jamaica would enable a better understanding of its scope, impacts, and determinants.

The study has several important limitations, mainly the lack of administrative registry data on health workers and migration specifically, and its cross-sectional nature. Nevertheless, the study’s key findings were validated repeatedly by diverse groups of key Jamaican stakeholders as being consistent with their experiences and providing an important evidence base on which to build stronger health workforce management and development strategies.

While many attempts have been made over the last few decades to address the problem of health worker migration from Jamaica, this study reveals that it remains very common. Perhaps more importantly, the study underscores how little is known about the impacts any of the efforts to better manage this migration have had. This study provides important evidence on the scope, drivers and impacts of migration of health workers from Jamaica, which can inform efforts by the MoH and its partners to better manage this phenomenon. It also highlights areas that merit further study, such as the various potential strategies suggested by participants for better managing health worker migration from Jamaica. Investigating, implementing, and evaluating these strategies will require ongoing collaboration between the MoH and these partners.

Finally, a potential outcome of the full, four-country study is that the collective findings from each participating country will help inform international dialogue and policies related to the ethics of health worker migration so that efforts to recruit healthcare professionals are negotiated with ‘source’ countries.

## Abbreviations

CARICOM: Caribbean Community and Common Market
KPH: Kingston Public Hospital
MoH: Ministry of Health
OECD: Organisation for Economic Co-operation and Development
PAHO: Pan American Health Organization
RHA: Regional Health Authority
